# Supplementary material for: ppGpp functions as an alarmone in metazoa
Source: Commun Biol. 2020 Nov 13;3:671. doi: 10.1038/s42003-020-01368-4 (PMC7666150; doi:10.1038/s42003-020-01368-4)
Supplement: Supplementary file 1 — Supplementary Information [file 42003_2020_1368_MOESM1_ESM.pdf]

Supplementary Information

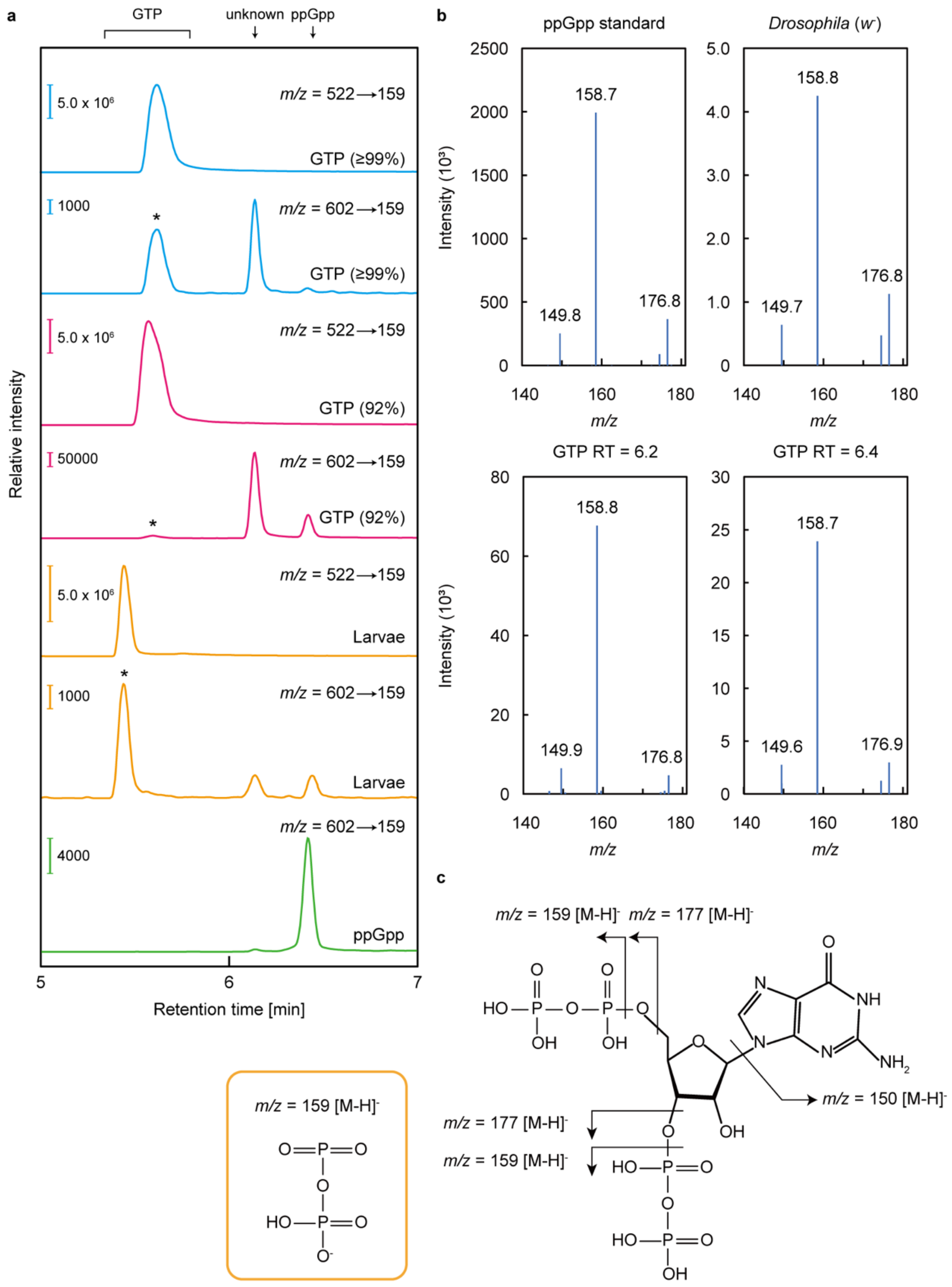

Supplementary Fig. 1: MS/MS chromatograms for GTP and ppGpp. a Comparison of MS/MS chromatograms for GTP (*m/z* = 522→159) and ppGpp (*m/z* = 602→159). GTP (92% or ≥99%) and ppGpp standards as well as the larvae extract were analyzed using the multiple reaction monitoring mode. The first quadrupole separated ppGpp (*m/z* = 602) or GTP (*m/z* = 522), and the second quadrupole separated pyrophosphate (*m/z* = 159) derived from GTP and/or ppGpp. *Multiple reaction monitoring–dependent cross talk from GTP. **b** MS spectra of product ions of the predicted ppGpp elusion peak of the ppGpp standard and the *Drosophila* larvae extract (upper), as well as those of the unknown molecule (RT = 6.2) and ppGpp (RT = 6.4) elusion peaks in the GTP standard (92%) (Bottom). RT, retention time. **c** Schematics of predicted fragmentation of ppGpp in the MS/MS analysis.

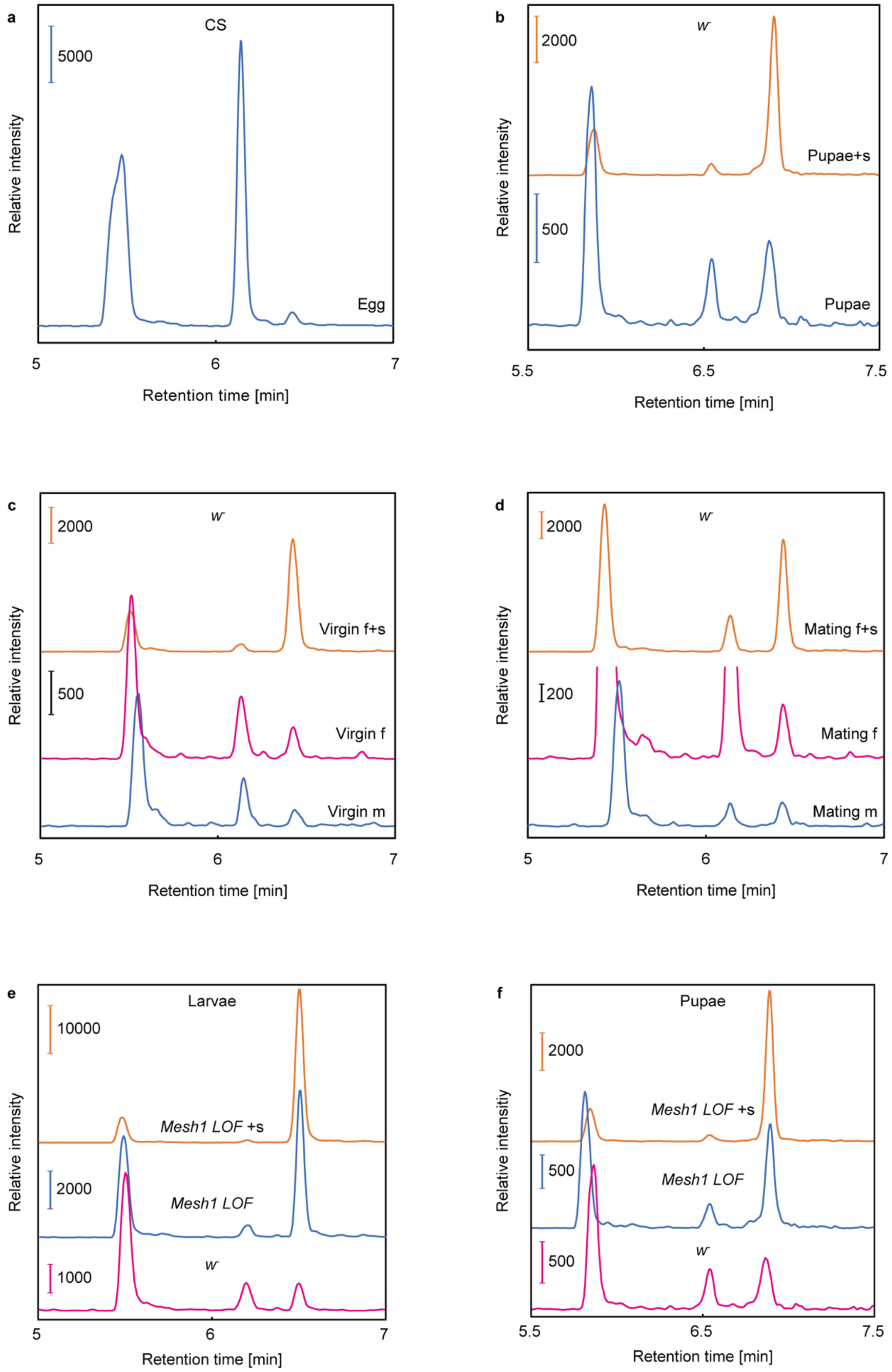

a
CS
5000
Egg
Relative intensity
5
6
7
Retention time [min]
b
w⁻
2000
500
Pupae+s
Pupae
Relative intensity
5.5
6.5
7.5
Retention time [min]
c
w⁻
2000
500
Virgin f+s
Virgin f
Virgin m
Relative intensity
5
6
7
Retention time [min]
d
w⁻
2000
200
Mating f+s
Mating f
Mating m
Relative intensity
5
6
7
Retention time [min]
e
Larvae
10000
2000
1000
Mesh1 LOF +s
Mesh1 LOF
w⁻
Relative intensity
5
6
7
Retention time [min]
f
Pupae
2000
500
500
Mesh1 LOF +s
Mesh1 LOF
w⁻
Relative intensitiy
5.5
6.5
7.5
Retention time [min]

Supplementary Fig. 2: Detection of ppGpp in various stages of *Drosophila*. Guanosine tetraphosphate–specific MS chromatograms of nucleotide pools extracted from CS eggs (**a**), *w*⁻ pupae (**b**), *w*⁻ virgin flies (male/female) (**c**), *w*⁻ mating flies (male/female) (**d**), *Mesh1 LOF* larvae (**e**), and *Mesh1 LOF* pupae (**f**). The symbol + denotes the exogenous addition of ppGpp standard to the extract.

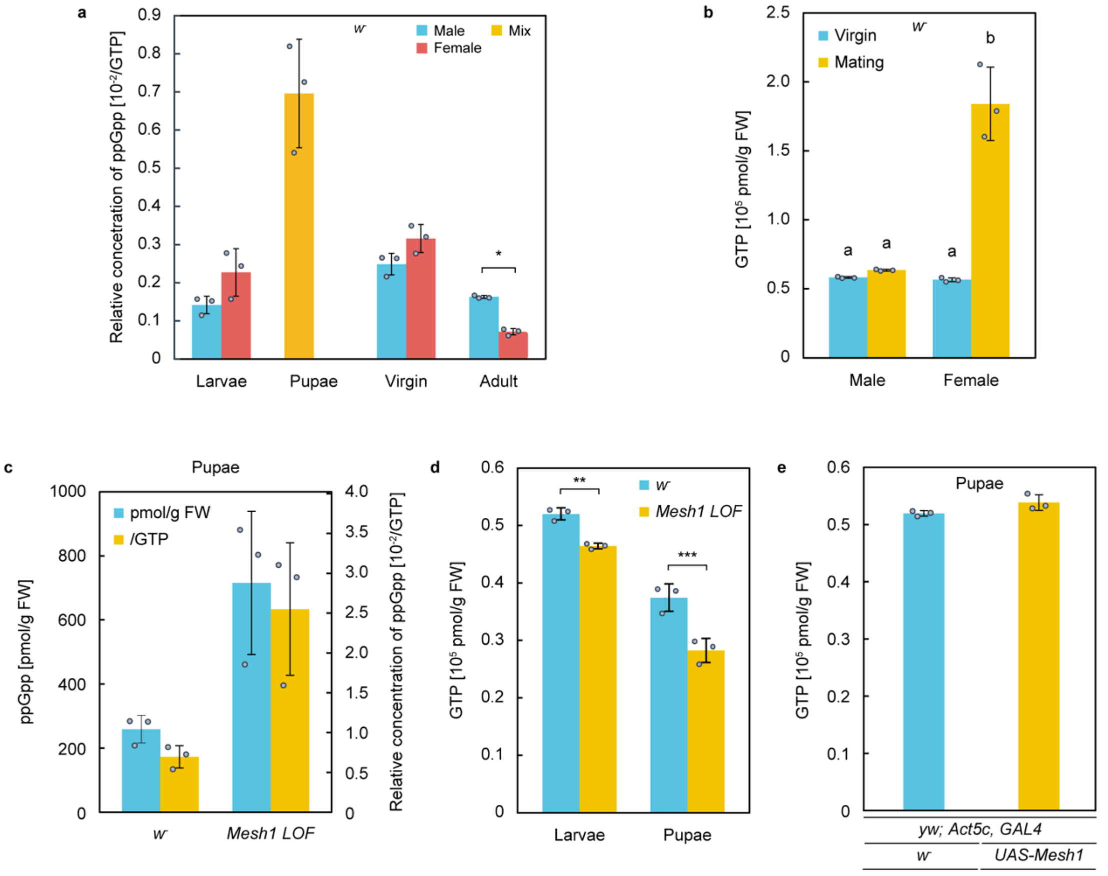

Supplementary Fig. 3: Concentrations of ppGpp and/or GTP in various stages of *Drosophila*. Various stages of development of *Drosophila w⁻*, *Mesh1 LOF*, and/or *Mesh1 GOF*. ppGpp levels per GTP in various stages of *w⁻* (**a**), GTP levels per FW *in male or female of w⁻* (**b**), ppGpp levels per FW or GTP *in Mesh1 LOF pupae* (**c**), GTP levels per FW in *Mesh1 LOF* larvae and pupae (**d**), and GTP levels per FW in *Mesh1 GOF* pupae (**e**). Values represent the mean ± S.D. (n =3 biologically independent samples). *p =0.0008, **p =0.0043, ***p =0.0121; two-sided Student's t-test. Different letters (a, b) indicate significant differences between groups (p < 0.05, Tukey's test). Male and female flies could not be distinguished at pupae stages, so they were sampled together.

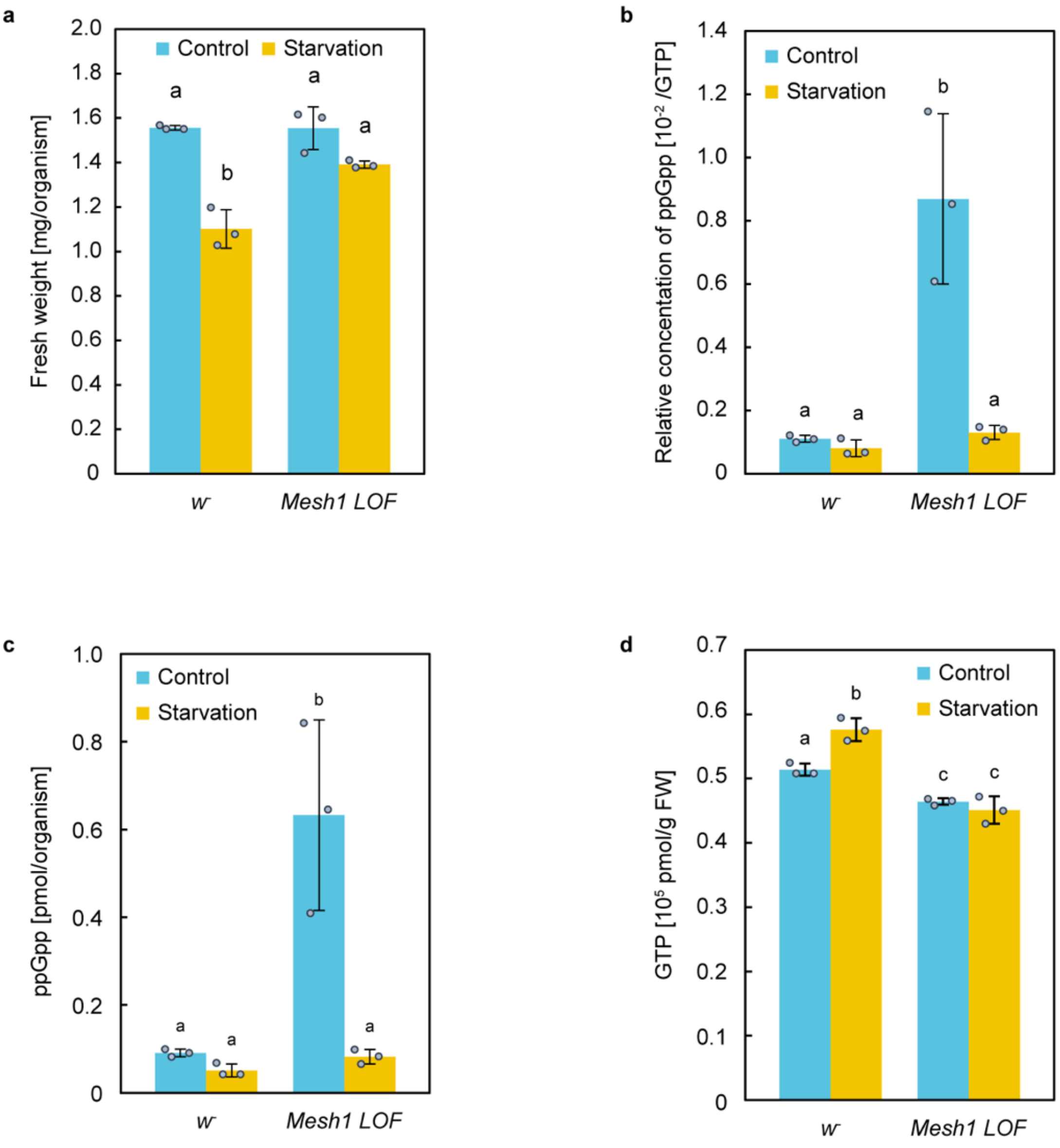

Supplementary Fig. 4: Starvation-inducible phenotypes of *Drosophila*. Fresh weight (FW) (**a**), ppGpp levels per GTP (**b**), ppGpp levels per organism (**c**), and GTP levels per FW (**d**) of w^{-} and *Mesh1 LOF*. Values represent the mean ± S.D. (n =3 biologically independent samples). Different letters (a-c) indicate significant differences between groups ($p < 0.05$, Tukey's test). Control, flies grown under non-starvation conditions.

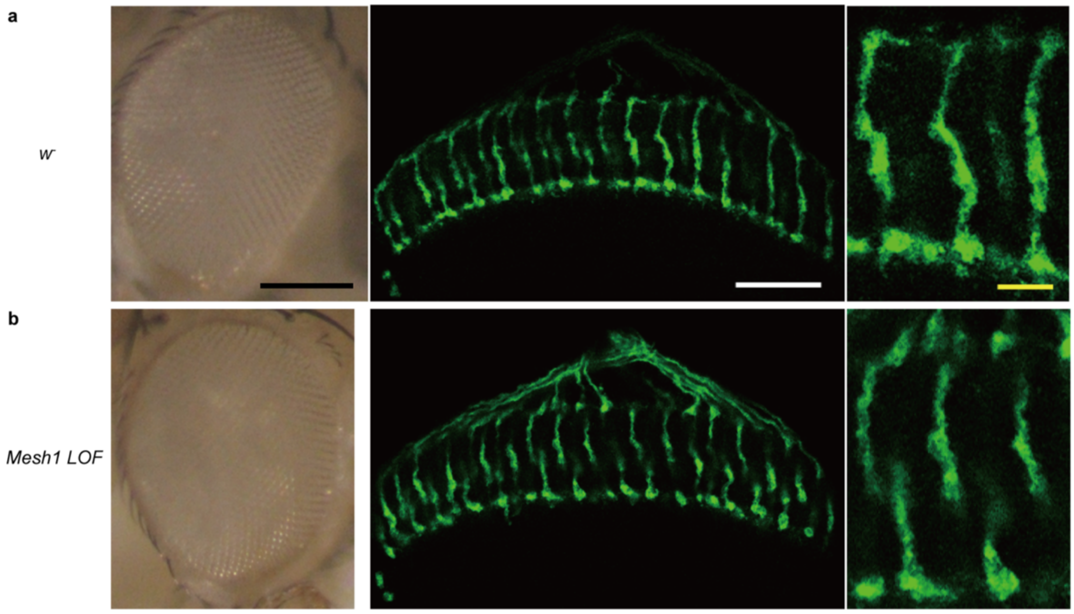

Supplementary Fig. 5: Effect of *Mesh1* mutation on eye development. Optical microscope images (left) and confocal images of dissected eyes (center and right) in w^{-} (**a**) and *Mesh1 LOF* (**b**). Axons were observed by immunostaining with the chaoptin-specific antibody. Observations were made one day after emergence. Black, white and yellow bar = 100 μm, 20 μm and 5 μm, respectively.

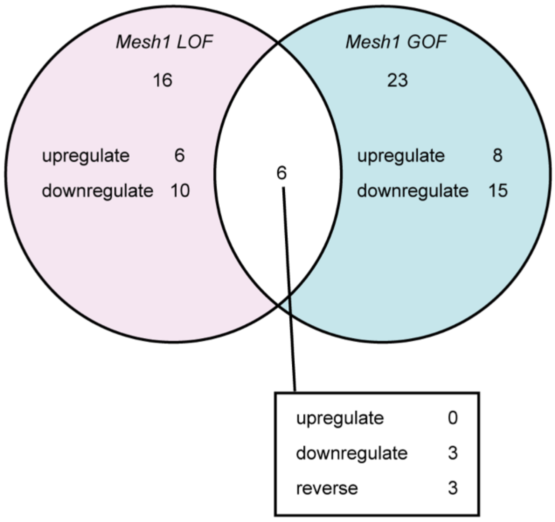

Supplementary Fig. 6: Venn diagram representing the number of significantly changed metabolites. Metabolites in *Mesh1 LOF* (left) and *Mesh1 GOF* (right) compared with those of controls. Reverse means that the metabolite changes were in the opposite direction (in "reverse" all the metabolites in *Mesh1 LOF* were upregulated, and all in *Mesh1 GOF* were downregulated).

Supplementary Table 1: ppGpp levels (per FW or GTP) in each stage.

Species	pmol/g FW	S.D.	(10^{-3})/GTP	S.D. (10^{-3})
Drosophila melanogaster				
**CS (WT)**				
Eggs	209.6	11.1	N.D.	N.D.
w^{-} **(WT)**				
Third instar larvae (male)	53.3	11.7	1.41	0.231
Third instar larvae (female)	63.5	15.6	2.27	0.624
Starved larvae	47.1	16.9	0.812	0.266
Pupae (day 2)	259	43.2	6.96	1.42
Virgin (male)	145	17.3	2.49	0.282
Virgin (female)	179	25.2	3.16	0.367
Mating adults (male, day 4)	103	2.44	1.63	0.0372
Mating adults (female, day 4)	129	3.49	0.71	0.083
Mesh1 LOF				
Third instar larvae	403	121	8.61	2.64
Starved larvae	59.1	11.3	1.31	0.23
Pupae (day 2)	716	223	25.5	8.31
yjbM OE (day 3 larvae)	45969	12183	185.3	96.6
Mesh1 GOF (day 2 pupae)	347	112	6.5	2.2
Mesh1 GOF control	652	294	12.6	5.7
Germ-free larvae	24.8	5.9	0.129	0.0155
**Cultured cells**				
HeLa	43.5	12.6	2.54	0.253
S2	N.D.	N.D.	2.74	0.78

Values represent the mean ± S.D. (*n* =3 biologically independent samples).

N.D., not determined.

Supplementary Table 2: Metabolites that were present in significantly different amounts in *Mesh1 LOF* compared with WT.

Annotation Name	*Mesh1 LOF*/WT	*p* value
4-Hydroxybenzoate	1.87	1.75×10^{-2}
Fumarate	1.45	5.00×10^{-2}
GABA	1.43	3.13×10^{-2}
Malate	1.4	2.98×10^{-3}
His	1.35	1.58×10^{-2}
CysSG	1.34	4.97×10^{-2}
Raffinose	1.31	2.47×10^{-2}
Methylneuraminate	1.27	2.69×10^{-2}
Carnitine	1.23	1.20×10^{-3}
HMG	0.77	9.56×10^{-3}
Arg	0.76	2.18×10^{-2}
Gly-Leu	0.64	1.50×10^{-2}
GlcNAc1P	0.64	8.40×10^{-4}
Lys	0.6	3.50×10^{-2}
Taurine	0.55	1.88×10^{-5}
NADP	0.5	4.96×10^{-2}
Cytosine	0.48	7.17×10^{-3}
Sedoheptulose 7-phosphate	0.44	3.23×10^{-3}
Deoxyguanosine	0.39	4.13×10^{-2}
Sarcosine	0.31	2.96×10^{-4}
Kynurenine	0.22	9.15×10^{-3}
1-Methyladenosine	0.12	9.53×10^{-5}

Third instar larvae grown in standard medium were used for metabolite analyses. For each metabolite, the relative increase or decrease and *p* value of the two-sided Student's *t*-test are indicated (*n* = 6 biologically independent samples). GABA: gamma-aminobutyric acid, CysSG: cysteineglutathione disulfide, HMG: β-hydroxy β-methylglutaryl-CoA, GlcNAc1P: N-acetyl-α-D-glucosamine 1-phosphate, NADP: nicotinamide adenine dinucleotide phosphate.

Supplementary Table 3： Metabolites that were present in significantly different amounts in *Mesh1 GOF* compared with WT.

Annotation Name	*Mesh1 GOF* /con.	*p* value
Adenosine	2.1	3.37×10^{-2}
Adenine	1.62	1.87×10^{-2}
CDP	1.62	2.25×10^{-2}
5-OxoPro	1.32	2.14×10^{-2}
Pyridoxamine5P	1.26	2.81×10^{-2}
CML	1.19	2.81×10^{-3}
PhenylP	1.17	1.66×10^{-2}
Pro	1.15	2.01×10^{-2}
3-MethylHis	0.86	4.23×10^{-2}
Tyr	0.84	6.30×10^{-4}
Deoxyuridine	0.79	4.72×10^{-4}
HomoPro	0.75	1.87×10^{-2}
Trp	0.74	2.03×10^{-2}
Malate	0.74	1.05×10^{-2}
Methylneuraminate	0.73	9.58×10^{-3}
Fumarate	0.73	1.02×10^{-2}
Fructose-Pro	0.72	5.95×10^{-4}
Fructose-Ala	0.71	1.34×10^{-3}
GlcNAc1P	0.71	3.63×10^{-4}
N8-Acetylspermidine	0.71	2.52×10^{-2}
Met	0.68	6.90×10^{-3}
Hypoxanthine	0.67	5.45×10^{-3}
Creatine	0.64	9.51×10^{-4}
Citrulline	0.57	3.01×10^{-3}
2-MethylSer	0.55	2.42×10^{-2}
Sarcosine	0.42	2.21×10^{-5}
Cystathionine	0.41	3.93×10^{-3}
MethylAla	0.25	2.12×10^{-5}
Kynurenine	0.23	3.83×10^{-3}

Third instar larvae grown in standard medium were used for metabolite analyses. For each metabolite, the relative increase or decrease and *p* value of the two-sided Student's *t*-test are indicated ($n = 6$ biologically independent samples). CDP: cytidine diphosphate, 5-

OxoPro: pyroglutamic acid, PhenylP: phenylephrine, CML carboxymethyl lysine, 3-MethylHis: 3-methylhistidine, HomoPro: homoproline, GlcNAc1P: N-acetyl-α-D-glucosamine 1-phosphate, 2-MethylSer: 2-methylserine, MethylAla: methylalanine.
