# Supplementary material for: ppGpp functions as an alarmone in metazoa
Source: Commun Biol. 2020 Nov 13;3:671. doi: 10.1038/s42003-020-01368-4 (PMC7666150; doi:10.1038/s42003-020-01368-4)
Supplement: Supplementary file 3 — Description of Additional Supplementary Files [file 42003_2020_1368_MOESM3_ESM.pdf]

Description of Additional Supplementary Files

Title: Supplemental Data file 1

Description: Original data sets of ppGpp and GTP quantification as well as metabolome analysis.
