# Supplementary material for: ppGpp functions as an alarmone in metazoa
Source: Commun Biol. 2020 Nov 13;3:671. doi: 10.1038/s42003-020-01368-4 (PMC7666150; doi:10.1038/s42003-020-01368-4)
Supplement: Supplementary file 4 — Reporting Summary [file 42003_2020_1368_MOESM4_ESM.pdf]

nature research

Corresponding author(s): Shinji Masuda

Last updated by author(s): Oct 6, 2020

Reporting Summary

Nature Research wishes to improve the reproducibility of the work that we publish. This form provides structure for consistency and transparency in reporting. For further information on Nature Research policies, see our Editorial Policies and the Editorial Policy Checklist.

Statistics

For all statistical analyses, confirm that the following items are present in the figure legend, table legend, main text, or Methods section.

n/a	Confirmed	
☐	☒	The exact sample size (*n*) for each experimental group/condition, given as a discrete number and unit of measurement
☐	☒	A statement on whether measurements were taken from distinct samples or whether the same sample was measured repeatedly
☐	☒	The statistical test(s) used AND whether they are one- or two-sided<br>*Only common tests should be described solely by name; describe more complex techniques in the Methods section.*
☒	☐	A description of all covariates tested
☒	☐	A description of any assumptions or corrections, such as tests of normality and adjustment for multiple comparisons
☒	☐	A full description of the statistical parameters including central tendency (e.g. means) or other basic estimates (e.g. regression coefficient) AND variation (e.g. standard deviation) or associated estimates of uncertainty (e.g. confidence intervals)
☐	☒	For null hypothesis testing, the test statistic (e.g. *F*, *t*, *r*) with confidence intervals, effect sizes, degrees of freedom and *P* value noted<br>*Give P values as exact values whenever suitable.*
☒	☐	For Bayesian analysis, information on the choice of priors and Markov chain Monte Carlo settings
☒	☐	For hierarchical and complex designs, identification of the appropriate level for tests and full reporting of outcomes
☒	☐	Estimates of effect sizes (e.g. Cohen's *d*, Pearson's *r*), indicating how they were calculated

Our web collection on statistics for biologists contains articles on many of the points above.

Software and code

Policy information about availability of computer code

Data collection	ppGpp and GTP quantification was achieved with Waters LC-MS/MS ACQUITY UPLC H-ClassSystem Driver Version 1.5. Immunohistochemistry and its imaging was achieved with Nikon C2+ confocal microscopes and processed with NIS-elements AR and Adobe Photoshop. The G1601BA 3D-CE ChemStation software for CE and G3335-64002 MH Workstation were used for metabolome analysis.
Data analysis	Turkey's test (MEPHAS: http://www.gen-info.osaka-u.ac.jp/MEPHAS/tukey-e.html), t-test (Excel)

For manuscripts utilizing custom algorithms or software that are central to the research but not yet described in published literature, software must be made available to editors and reviewers. We strongly encourage code deposition in a community repository (e.g. GitHub). See the Nature Research guidelines for submitting code & software for further information.

Data

Policy information about availability of data

All manuscripts must include a data availability statement. This statement should provide the following information, where applicable:

- Accession codes, unique identifiers, or web links for publicly available datasets
- A list of figures that have associated raw data
- A description of any restrictions on data availability

The dataset generated and analyzed in the current study is available as Supplementary Data file 1.

Field-specific reporting

Please select the one below that is the best fit for your research. If you are not sure, read the appropriate sections before making your selection.

[x] Life sciences   [ ] Behavioural & social sciences   [ ] Ecological, evolutionary & environmental sciences

For a reference copy of the document with all sections, see nature.com/documents/nr-reporting-summary-flat.pdf

Life sciences study design

All studies must disclose on these points even when the disclosure is negative.

Sample size	Samples sizes were determined based on our previous studies (e.g. Ref. 19, 32). A minimum of n = 3 individuals was used.
Data exclusions	We have not excluded any data.
Replication	All the data presented in the study were reproduced a minimum of three times.
Randomization	We provided samples randomly for quantification of experimental groups.
Blinding	Investigations were blinded during data collection and analysis

Reporting for specific materials, systems and methods

We require information from authors about some types of materials, experimental systems and methods used in many studies. Here, indicate whether each material, system or method listed is relevant to your study. If you are not sure if a list item applies to your research, read the appropriate section before selecting a response.

Materials & experimental systems

n/a	Involved in the study	
[ ]	[x]	Antibodies
[ ]	[x]	Eukaryotic cell lines
[x]	[ ]	Palaeontology and archaeology
[ ]	[x]	Animals and other organisms
[x]	[ ]	Human research participants
[x]	[ ]	Clinical data
[x]	[ ]	Dual use research of concern

Methods

n/a	Involved in the study	
[x]	[ ]	ChIP-seq
[x]	[ ]	Flow cytometry
[x]	[ ]	MRI-based neuroimaging

Antibodies

Antibodies used	anti-chaoptin (mAb24B10, DSHB), Alexa488 (Life Technologies)
Validation	The antibodies were validated and used previously (Ref. 59).

Eukaryotic cell lines

Policy information about cell lines

Cell line source(s)	HeLa and PEAKrapid (American Type Culture Collection), Drosophila S2 (GIBCO, R690-07)
Authentication	HeLa and S2 cells were recently obtained from ATCC and GIBCO, who authenticated the products. PEAKrapid was obtained from ATCC 20 years ago, and we have not provided Authentication by ourselves.
Mycoplasma contamination	We confirmed that all cell lines tested negative for Mycoplasma contamination
Commonly misidentified lines (See ICLAC register)	N/A

Animals and other organisms

Policy information about studies involving animals; ARRIVE guidelines recommended for reporting animal research

Laboratory animals	Drosophylla

Wild animals	N/A
Field-collected samples	N/A
Ethics oversight	We do not have any restrictions for Drosophylla research

Note that full information on the approval of the study protocol must also be provided in the manuscript.
